# Analysis of Maternal Postnatal Depression, Socioeconomic Factors, and Offspring Internalizing Symptoms in a Longitudinal Cohort in South Africa

**DOI:** 10.1001/jamanetworkopen.2021.21667

**Published:** 2021-08-19

**Authors:** Massimiliano Orri, Sahba Besharati, Marilyn N. Ahun, Linda M. Richter

**Affiliations:** 1 McGill Group for Suicide Studies, Douglas Mental Health University Institute, Department of Psychiatry, McGill University, Montréal, Québec, Canada; 2 Bordeaux Population Health Research Centre, Inserm U1219, University of Bordeaux, Bordeaux, France; 3 Department of Psychology, University of the Witwatersrand School of Human and Community Development, Johannesburg, South Africa; 4 Department of Social and Preventive Medicine, Université de Montréal School of Public Health, Montréal, Québec, Canada; 5 Department of Science and Innovation-National Research Foundation Centre of Excellence in Human Development, University of the Witwatersrand, Johannesburg, South Africa

## Abstract

**Importance:**

Few studies from low-and-middle income countries have investigated long-term associations between maternal postnatal depression and offspring internalizing (ie, depressive and anxiety) symptoms, and none have investigated interactions in this association.

**Objective:**

To investigate the association between maternal postnatal depression and offspring internalizing symptoms from adolescence to adulthood and the interaction with exposure to socioeconomic adversity and with the child’s sex.

**Design, Setting, and Participants:**

This secondary analysis used data from Birth to Twenty Plus (BT20+), a prospective birth cohort study of children born in Soweto, South Africa, and followed up until age 28 years. Data were collected from 1990 to 2018, and data were analyzed for this study from February 16 through December 15, 2020.

**Exposures:**

Maternal postnatal depression self-reported by mothers 6 months after childbirth.

**Main Outcomes and Measures:**

The main outcome was offspring internalizing symptoms, assessed at offspring ages 14 years, 22 years, and 28 years and modeled longitudinally. Participants with the highest probability of experiencing high internalizing symptoms (ie, those in the top 20% of the distribution) from age 14 to 28 years were categorized as belonging to the high internalizing symptoms trajectory (vs the low trajectory). Socioeconomic adversity was measured with an index (continuous variable) including low maternal education, household crowding, low assets, and low maternal age. This variable was further stratified into more than 1 SD above the mean index, more than 1 SD below the mean index, and from 1 SD below to 1 SD above the mean index to conduct subgroup analyses. Associations were investigated using multivariable regression models.

**Results:**

Among 1087 participants born in Soweto, South Africa (543 [50.0%] male participants; 544 [50.0%] female participants), 118 individuals (10.8%) showed a high trajectory of internalizing symptoms from age 14 to 28 years vs 969 individuals (89.1%) with a low trajectory. Children exposed to maternal postnatal depression had statistically significantly increased odds of following the high trajectory (adjusted odds ratio [aOR] per 1-SD increase in maternal postnatal depression, 1.20; 95% CI, 1.02-1.41). This increase in odds differed by exposure to socioeconomic adversity and by child sex: for male participants, the increase in odds was greater in a context of higher vs lower socioeconomic adversity (eg, for >1 SD above the mean: aOR, 3.28; 95% CI, 1.06-10.14 vs for >1 SD below the mean: aOR, 0.98; 95% CI, 0.64-1.50; *P* for interaction = .12), while for female participants, the increase in odds was greater in a context of lower vs higher socioeconomic adversity (eg, for >1 SD below the mean: aOR, 1.82; 95 % CI, 1.12-2.98 vs for >1 SD above the mean: aOR, 0.59; 95 % CI, 0.30-1.17; *P* for interaction = .002) (*P* for 3-way interaction = .003).

**Conclusions and Relevance:**

This study found that postnatal depression was associated with higher odds of persistently increased internalizing symptoms among offspring from adolescence to adulthood in a middle-income country, with variation by socioeconomic adversity and sex. These findings suggest that better understanding of these associations is needed to implement targeted interventions and maximize the impact of public health initiatives aimed at breaking the intergenerational transmission of mental health problems.

## Key Points

**Question:**

Is there an interaction with socioeconomic adversity in the association between postnatal maternal depression and offspring internalizing symptoms in low-and-middle income countries (LMICs)?

**Findings:**

In this cohort study among 1087 individuals born in Soweto, South Africa, maternal depression 6 months after childbirth was associated with increased odds of internalizing symptoms from adolescence to adulthood. For male participants, the increase in odds was greater in a context of higher vs lower socioeconomic adversity, while for female participants, the increase in odds was greater in a context of lower vs higher socioeconomic adversity.

**Meaning:**

These findings suggest that prevention of postnatal depression in LMIC settings may be associated with decreases in the prevalence of psychological problems in the next generation; such prevention may be maximized by considering individual differences by sex and exposure to socioeconomic adversity.

## Teaser

This cohort study examines the association between maternal postnatal depression and offspring internalizing symptoms and the interaction of socioeconomic adversity and child sex with this association.

## Introduction

The early home environment is associated with mental health outcomes across an individual’s lifespan.^1^ Specifically, exposure to maternal depression has consistently been associated with offspring internalizing depressive and anxiety symptoms.^2,3^ The prevalence of maternal perinatal (ie, antenatal or postnatal) depression is 11% to 13% among women in high-income countries^4^ (HICs; according to World Bank definitions).^5^ Prevalence rates are increased for women in low and middle income countries (LMICs; according to World Bank definitions), with a 2016 systematic review^1^ reporting that 1 in 4 women presented with antenatal depression and 1 in 5 presented with postnatal depression. Despite increased rates of perinatal depression in LMICs,^6^ it is a neglected area of research, with limited evidence on the short-term associations and long-term associations with child outcomes. Given this lack of study, women’s and children’s mental health problems within these contexts are largely unrecognized and untreated.^1,7^ Indeed, the context within which perinatal depression and internalizing symptoms occur differ in LMICs, where children are more likely to be exposed to many risk factors, including poverty and high rates of violence, compared with HICs. Context-specific studies are therefore key to identifying and targeting potential interventions to prevent and reduce internalizing symptoms among offspring exposed to perinatal depression, as well as to ensure that interventions are tailored to the local context.

In HICs, studies have found that perinatal depression is associated with internalizing symptoms across childhood and adolescence^8,9^ and that there is an interaction with individual and environmental characteristics for this association. At the individual level, 2 studies^3,8^ found a greater increase in internalizing symptoms with perinatal depression among girls, while others find no evidence of sex differences. At the environmental level, studies report an interaction with socioeconomic adversity whereby children exposed to perinatal depression and low socioeconomic adversity have better mental health outcomes compared with children exposed to cumulative adversity (ie, perinatal depression and high socioeconomic adversity, including low levels of parental education and financial support).^2,3,10^ Interactions with sex or socioeconomic adversity could suggest that different intervention approaches may be required to mitigate the association of maternal depression with child outcomes in boys vs girls and families from lower vs higher socioeconomic adversity contexts.^10^ For example, in the Avon Longitudinal Study of Parents and Children in the UK,^11^ for children exposed to the same level of perinatal depression, those experiencing greater socioeconomic adversity were more likely to experience increased rates of internalizing symptoms. That study did not explore the interaction with child’s sex. In the handful of studies that have been conducted in LMICs,^7,12,13,14,15,16,17,18^ the pattern of associations between perinatal depression and children’s internalizing symptoms is generally similar to that found in HICs, including mixed evidence on the interaction with child’s sex. However, to our knowledge, no study to date has examined the interaction of socioeconomic adversity with maternal depression in the association with offspring internalizing symptoms in LMICs. This is acknowledged as one of the largest gaps in the current literature on perinatal depression and child outcomes in LMICs.^1,10^

Studies suggest that the pattern of associations between socioeconomic factors and mental health in HICs is not the same as in LMICs. For example, a cross-national comparative study^19^ of adults in HICs and LMICs found conflicting results in investigating the association between socioeconomic adversity and mental health. These findings highlight the importance of context-specific studies to understand how the interaction between socioeconomic adversities and mental health plays out in different sociocultural contexts.^10^

In this study, we examined the interaction of socioeconomic adversity and postnatal depression in the association with offspring internalizing symptoms across adolescence and early adulthood in male and female participants in South Africa. Given mixed evidence from prior studies in LMICs, we had no a priori hypotheses concerning the interaction of child’s sex and socioeconomic adversity with postnatal depression in the association with offspring internalizing symptoms.

## Methods

Ethical approval for this cohort study was obtained from the Committee for Research on Human Subjects at the University of the Witwatersrand in South Africa, and written informed consent was obtained from all participants. We adhered to the Strengthening the Reporting of Observational Studies in Epidemiology (STROBE) reporting guideline for standard reporting in cohort studies.

### Participants

This study draws on secondary data from the Birth to Twenty Plus (BT20+) study, a prospective birth cohort based in Soweto, a historically informal settlement in Johannesburg, South Africa.^20^ The cohort enrolled all singleton children born during a 7-week period in 1990, with the aim of describing the associations between rapid urbanization and the physical and psychosocial development of children.^20^ Women were pregnant and gave birth during an extremely volatile and violent time in South Africa’s history, both politically and socially. A sample of 3273 women was recruited, and they and their children have been followed up more than 22 times to offspring age 28 years (data collection from child’s birth in 1990 to 2018). Data have been collected on social and economic circumstances, family relationships, growth and health outcomes, and schooling and employment. Consensual agreement on the phrasing of questions was reached when different languages were required, with isiZulu, Sesotho, and English being the most commonly used languages. From participants’ age 15 years, data have been collected through audio-assisted computer methods and tablets. The sample in this study included 1087 participants with data on postnatal depression and offspring internalizing symptoms (≥1 measure collected per participant from ages 14-28 years). Participants who had data on postnatal depression alone or offspring internalizing symptoms alone were excluded from analyses. Because the analytical sample differed from the original cohort on several variables (ie, maternal age, schooling, household crowding, and family assets), inverse probability weighting was used in all analyses.

### Assessment of Postnatal Depression

Postnatal maternal depression was assessed when children were age 6 months using the Pitt inventory.^21^ This measure has been previously used in numerous studies in HICs and LMICs, including South Africa, for a period extending 20 years.^18,22,23^ It correlates highly (ρ = 0.67) with other criterion standard measures of postnatal depression, such as the Edinburgh Postnatal Depression Scale.^24^ This validated measure consists of 24 items (Cronbach α, 0.85) assessing current feelings and changes in mood. The inventory is scored on a 3-point scale (1 for yes, 0 for no, and missing for I don’t know). The total score was computed (range 0-24) and standardized.

### Assessment of Offspring Internalizing Symptoms

Internalizing symptoms were assessed among children at ages 14 years, 22 years, and 28 years using validated self-report questionnaires, including common and age-appropriate anxiety and depression symptoms. At children’s age 14 years, we used 24 items from the Youth Self Report’s internalizing scale (Cronbach α, 0.86), a measure specifically designed for adolescents and developed by the Achenbach System of Empirically Based Assessment.^25^ It has strong psychometric properties gathered from diverse cultural settings,^26^ with the validity of the measure confirmed across multilingual and multicultural environments,^27,28^ including South Africa.^29^ The measure includes items such as “I am unhappy,” “I worry a lot,” and “I have headaches.” Items were answered on a 3-point scale (ie, 0 for not true, 1 for somewhat or sometimes true, and 2 for very or often true) in reference to the past 6 months. At participants’ age 22 years, we used 21 items from the General Health Questionnaire (GHQ-28; Cronbach α, 0.93)^30^ somatic, anxiety and insomnia, and depression sections, including items such as “lost much sleep over worry,” “been feeling nervous or strung up all the time,” and “felt that life is entirely worthless.” Items were answered on a 4-point scale (ie, 0 for not at all, 1 for no more than usual, 2 for rather more than usual, and 3 for much more than usual) with reference to current or recent feelings. The GHQ-28 is associated with other widely used measures of mental health cross-culturally in HICs and LMICs^31^ and has been used previously in South Africa.^32^ At participants’ age 28 years, we used the World Health Organization (WHO) Self Reporting Questionnaire,^33^ which shows strong validity compared with other commonly used measures of adult mental health, including in South African settings.^34,35^ The scale includes binary items (ie, yes and no) assessing the presence of 20 symptoms experienced during the previous 30 days, such as, “Do you often have headaches?”, “Do you feel nervous, tense, or worried?”, and “Do you sleep badly?” (Cronbach α, 0.93). Given that these 3 measures have different scales, we dichotomized the measures and considered participants scoring at the top 20% of symptoms within each measure as having high levels of internalizing symptoms. This enabled us to harmonize the 3 measures and model them longitudinally, as described in a later section.

### Socioeconomic Adversity

Drawing on Trude et al,^36^ socioeconomic adversity in the early life environment was measured using 4 indicators: (1) poverty (measured as being below the third wealth quintiles derived from a site-specific list of assets [ie, television, refrigerator, car, washing machine, and phone] according to the widely used and culturally validated methodology of Filmer and Pritchett^37,38,39^), (2) low maternal education (ie, ≤the third quartile of the distribution), (3) low maternal age (ie, <age 18 years at childbirth), and (4) household crowding (ie, >3 people per room). These cutoffs are based on international standards determined by WHO.^40^ The socioeconomic adversity index was calculated using a factor analysis model and standardized (ie, mean [SD], 0 [1]) (eTable 1 in the Supplement). Adversity was stratified as high and low (ie, those >1 SD above or >1 SD below the mean index, respectively), with individuals from 1 SD below to 1 SD above the mean as the reference group.

### Statistical Analysis

We used group-based trajectory modeling to identify groups of children following distinct developmental trajectories of internalizing symptoms from adolescence to adulthood.^41^ We modeled the probability of having high internalizing symptoms (ie, scoring in the top 20% of scores) at each point. The advantage of this method is that it allows one to identify individuals following trajectories of internalizing symptoms within a population without establishing arbitrary cutoffs. Additionally, this method relies on maximum likelihood estimation, enabling us to use all available information in the data instead of excluding participants with missing data.^41^ We therefore used data from all participants who reported internalizing symptoms on at least 1 occasion. We also conducted sensitivity analyses in which trajectories were estimated for participants with data available in 2 of 3 assessments.

We used logistic regression analysis to estimate the association between postnatal depression and trajectories of offspring internalizing symptoms. To investigate differential odds increases between postnatal depression and offspring internalizing symptoms by socioeconomic adversity, we tested the interaction between postnatal depression and socioeconomic adversity index score. All models were adjusted for child sex, birth weight (ie, <2.5 kg vs ≥2.5 kg), and birth order (ie, first, second, third or later born). Additionally, we systematically tested the interaction with child sex to investigate whether odds changes were similar for male participants vs female participants.

*P* values were evaluated using 2-sided 2-sample *t* tests and χ^2^ tests, and significance was set at *P* < .05. Data were analyzed using R statistical software version 3.6 (R Project for Statistical Computing) from February 16 through December 15, 2020.

## Results

Among 1087 mother-child dyads, whose characteristics are presented in the Table, there were 544 (50.0%) girls and 543 (50.0%) boys. A higher percentage of boys were born to a mother aged less than 18 years (43 boys [7.9%] vs 39 girls [7.2%]; *P* = .02). There were no other statistically significant differences in sample characteristics by sex. Across male and female participants, similar mean (SD) levels of postnatal depression scores (0.001 [1.032] vs −0.001 [0.971]) and socioeconomic adversity scores (−0.002 [0.618] vs −0.026 [0.601]) were observed. Features of the socioeconomic environment were not associated with maternal postnatal depression. Internalizing symptoms showed consistency across time: adolescents with high symptom levels at age 14 years were 2-fold as likely to have high symptom levels at age 22 years (odds ratio [OR], 2.34; 95% CI, 1.75-3.13) and age 28 years (OR, 2.03; 95% CI, 1.48-2.80), and young adults with high symptoms at age 22 years were more than 3-fold as likely to have high symptoms at age 28 years (OR, 3.78; 95% CI, 2.92-4.89).

**Table.** Sociodemographic Characteristics of Study Population

Characteristic^a^	No. (%)			*P* value
	Total (N = 1087)	Male offspring (n = 543)	Female offspring (n = 544)	
Child characteristic				
Low birth weight (<2500 g)	106 (9.8)	45 (8.3)	61 (11.2)	.13
Birth order				
First	438 (40.3)	214 (39.4)	224 (41.2)	.83
Second	329 (30.3)	166 (30.6)	163 (30.0)	
≥Third	320 (29.4)	163 (30.0)	157 (28.9)	
Socioeconomic environment				
Poverty	507 (46.6)	273 (50.3)	234 (43.0)	.72
Maternal low education	611 (57.1)	304 (56.8)	307 (57.4)	.90
Maternal age at childbirth <18 y	82 (7.5)	43 (7.9)	39 (7.2)	.02
High crowding (>3 people/room)	310 (33.3)	218 (46.7)	199 (42.9)	.27
Socioeconomic adversity index score, mean (SD)	0 (1.00)	0.02 (1.00)	−0.02 (1.00)	.45

^a^ Characteristics were measured at enrollment into the cohort.

**Figure 1.**
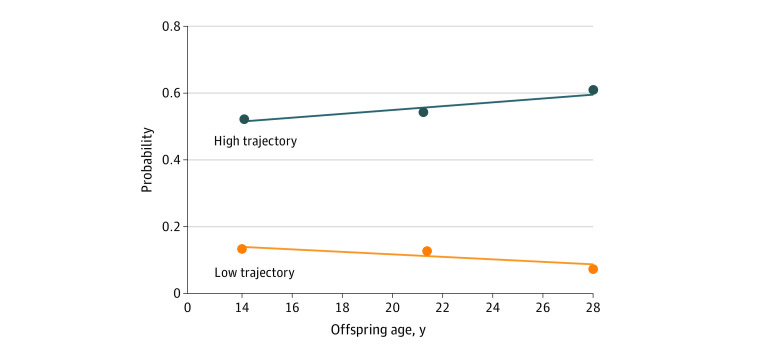
Trajectories of Internalizing Symptoms Trajectories of internalizing symptoms are shown from age 14 years through 22 to 28 years, estimated using group-based trajectory modeling.

Group-based trajectory modeling identified 2 trajectories (Figure 1): a high internalizing symptoms trajectory, clustering 118 individuals (10.8%) with high levels of internalizing symptoms from adolescence to adulthood, and a low internalizing symptoms trajectory, clustering 969 individuals (89.1%) with low levels of internalizing symptoms in this period. The selected model provided the best fit in terms of classification accuracy, as assessed by the mean posterior probability of class membership, entropy (0.83, with values >0.70 indicating good classification accuracy), and best fit compared with models with 1 or more than 2 trajectories, as assessed with the bayesian information criterion (BIC; best fitting model is the 1 minimizing BIC; 1-trajectory model: BIC = −1209.58; 2-trajectory model: BIC = −1188.49; 3-trajectory model: BIC = −1196.71; 4-trajectory model: BIC = −1208.40). The distribution of male and female participants differed by trajectory, with an overrepresentation of female participants in the high internalizing symptoms trajectory (89 female participants [74.4%]) compared with the low trajectory (455 female participants [47.0%]). The difference in mean (SD) socioeconomic adversity between participants in high vs low internalizing symptoms trajectories was larger for male participants (0.32 [1.00] vs −0.13 [1.00]) compared with female participants (0.04 [1.00] vs −0.06 [1.00]). However, similar mean (SD) maternal postnatal depression levels were found for participants in the high vs low internalizing symptoms trajectories among male participants (0.20 [0.92] vs 0.05 [1.00]) and female participants (0.15 [1.00] vs 0.02 [1.00]).

The adjusted OR (aOR) for the association between postnatal depression and high offspring trajectories of internalizing symptoms was 1.20 per 1-SD increase in postnatal depression (95 % CI, 1.02-1.41). This suggests that children of mothers with increased postnatal depression symptoms were more likely to follow the trajectory with high internalizing symptoms compared with children of mothers with low postnatal depression symptoms (ie, 20% higher odds for each 1-SD increase in postnatal depression).

**Figure 2.**
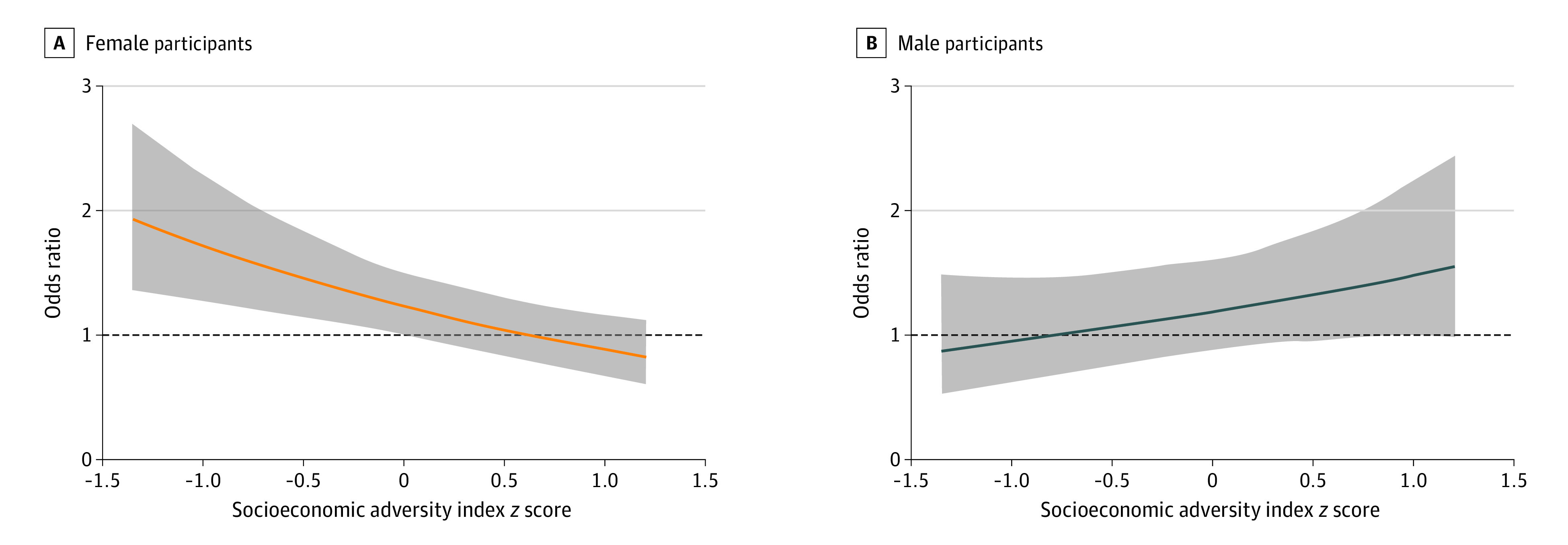
Interaction Between Postnatal Depression and Socioeconomic Adversity in the Association With Offspring Internalizing Symptoms For female participants, the increase in odds was greater in contexts of lower socioeconomic adversity, and for male participants, the increase in odds was greater in contexts of higher socioeconomic adversity.

In interaction analyses, this increase in odds was not homogenous across the sample but varied by level of socioeconomic adversity and child sex (3-way interaction: log[aOR], −0.57; SE, 0.19; *P* = .003). See eTable 2 and eFigure 1 in the Supplement for marginal predicted probabilities and eFigure 2 in the Supplement for interactions using each indicator of the socioeconomic adversity index. As represented in Figure 2, we found that, for female participants, the increase in odds of belonging to the high trajectory of offspring internalizing symptoms per 1-SD increase in maternal postnatal depression was greater among individuals exposed to lower levels of socioeconomic adversity compared with those exposed to higher levels (2-way interaction between maternal depression and socioeconomic adversity index among female participants: log[aOR] = −0.32, SE = 0.10; *P* = .002), while the opposite trend was observed for male participants (2-way interaction between maternal depression and socioeconomic adversity index among male participants: log[aOR] = 0.25; SE = 0.16; *P* = .12).

**Figure 3.**
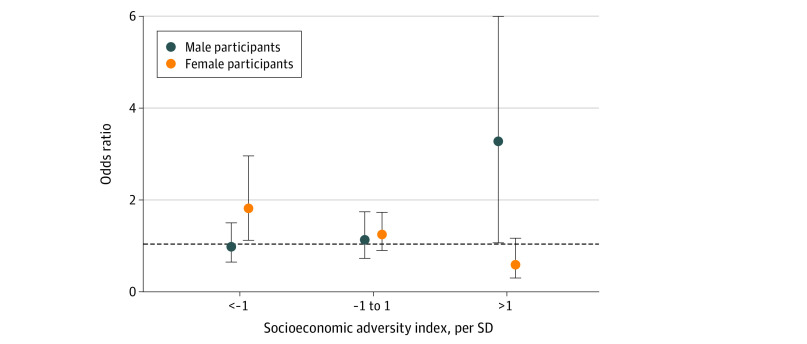
Association Between Maternal Postnatal Depression and Offspring Internalizing Symptoms by Socioeconomic Adversity Index Participants were stratified by socioeconomic adversity index in 3 groups: those with scores 1 SD below the mean, from −1 SD below the mean to and 1 SD above the mean, and greater than 1 SD above the mean. For female participants, the increase in odds was greater in contexts of low socioeconomic adversity, while for male participants, the increase in odds was greater in contexts of high socioeconomic adversity. The upper bound of the CI for male participants in the greater than 1 SD above the mean category (ie, 10.14) has been cut to 6 for visual purposes.

We conducted stratified analyses by levels of socioeconomic adversity index score to better understand the increase in odds of the high trajectory of internalizing symptoms associated with each 1-SD increase in postnatal depression. As shown in Figure 3, the increase in odds was greater for the lowest stratum of socioeconomic adversity (ie, >1 SD below the mean of the index) vs the highest stratum (ie, >1 SD above the mean of the index) among female participants (aOR, 1.82; 95% CI, 1.12-2.98 vs aOR, 0.59; 95% CI, 0.30-1.17) but not among male participants. In the highest stratum of socioeconomic adversity compared with the lowest stratum, there was a greater increase in odds among male participants (aOR, 3.28; 95% CI, 1.06-10.14 vs aOR, 0.98; 95 % CI, 0.64-1.50) but not among female participants.

## Discussion

To our knowledge, this cohort study is the first study to investigate the association between exposure to postnatal depression and offspring internalizing symptoms across adolescence and adulthood in the South African context. We found that exposure to postnatal depression in the first 6 months of life was associated with internalizing symptoms through adolescence and young adulthood up to age 28 years. Importantly, we found an interaction of child sex and of socioeconomic adversity with maternal depression in this association. Specifically, we found that, for male participants, the increase in odds of belonging to the high offspring internalizing symptoms trajectory when the mother had higher postnatal depression was greater in a context of higher compared with lower socioeconomic adversity; conversely, for female participants, the increase in odds was greater in a context of lower compared with higher socioeconomic adversity.

Prior studies in LMICs found that exposure to maternal depression early in life was associated with negative socioemotional outcomes during childhood.^7,42,43,44^ This includes 2 previous studies using BT20+ data, which reported that children exposed to postnatal depression (measured at child’s age 6 months) were more likely to experience psychological distress, inclusive of internalizing symptoms, in early childhood (ie, age 2 years)^13^ and middle childhood (ie, age 10 years)^18^ compared with those unexposed as infants. Therefore, our findings extend current knowledge by showing that the association between postnatal depression and internalizing symptoms is discernible during the first 3 decades of life. To date, this is 1 of the longest follow-up studies of this kind and the longest follow up in a LMIC. This finding is in line with previous studies^11,15,17,45,46,47,48,49,50^ in HICs and LMICs reporting a persistent association between maternal depression and offspring mental health in adolescence and adulthood. The Avon Longitudinal Study of Parents and Children in the UK,^11^ which was based on population samples, reported associations similar in size to those reported in our study.

Several mechanisms may explain the observed associations.^3^ From a biological perspective, genetic liability for mental health problems may account for the heightened vulnerability to internalizing symptoms among offspring of depressed mothers.^51^ Other biological mechanisms that may explain the association between postnatal depression and offspring outcomes include epigenetic modifications of gene expression and changes in the glucocorticoid, oxytocin, estrogen, and immune systems.^51^ From an environmental point of view, postnatal depression has been found to be associated with changes in maternal responsiveness toward her child, impaired ability to respond adequately to infant cues, inhibited maternal support and protection, and reduced maternal perceptions of self-efficacy.^10,52,53^ All of these outcomes have the potential to compromise the quality of parenting, as shown in a recent meta-analysis that found an interaction between maternal parenting behaviors and the association between perinatal depression and mental health outcomes across childhood and adolescence.^53^ Maternal mental health problems have also been associated with increased risk of family conflict and domestic violence, which, independently and jointly, are risk factors associated with poor mental health among offspring.^54^ Additionally, there is evidence that continued exposure to maternal depression during childhood, as opposed to limited or intermittent exposure, is associated with poor outcomes among offspring.^55,56^ It is worth noting that evidence for such mechanisms is available mainly for short-term associations and in HIC settings. Future studies should therefore further clarify whether and how these mechanisms explain associations that persist until adulthood in LMICs.^10^

In line with previous studies, we found an interaction between exposure to socioeconomic adversity, which is associated with parental mental health problems and limitations in a parent’s ability to provide for their child,^3,53^ and postnatal depression in the association with offspring internalizing symptoms. However, contrary to results from previous studies,^11,47^ which did not test for or did not observe sex differences, we found that the interaction was different among male participants (who had greater increases in odds of internalizing symptoms if exposed to maternal depression in a higher socioeconomic adversity context) vs female participants (who had greater increases in odds of internalizing symptoms if exposed to maternal depression in a lower socioeconomic adversity context). Understanding such differential interactions by sex, socioeconomic adversity, and maternal postnatal depression in South Africa would require additional investigations, such as qualitative in-depth studies considering social and cultural contexts in which children develop. Factors that may be associated with these outcomes include childcare practices (eg, mothers with very low levels of education may be more dependent on and receive help with raising their child from members of the extended family, including informal nonparental care from family members,^57^ which may be associated with decreased exposure to postnatal depression for those children). Additional factors may include the societal value of having a male or female child (including family expectations for child education and future social roles)^58^ and sex differences in reactivity to stress during childhood.^59^ Shedding light on how socioeconomic variations and individual characteristics interact in the association of postnatal depression with outcomes among children is a key step toward implementing personalized interventions and maximizing the impact of public health policy.

### Limitations

This study has several limitations. First, attrition was substantial, although comparable with that of other longitudinal cohort studies.^60,61^ Attrition in the BT20+, which has been described elsewhere,^20^ is due to a combination of factors, including temporary and permanent migration, which may compromise generalizability of the findings to all of South Africa’s diverse population and to people living at the higher end of the socioeconomic scale. To address biases due to differential attrition, we used inverse probability weighting, but many factors potentially associated with attrition may not have been considered. Furthermore, the analysis sample was not representative of the country’s general population, as is the case with other longitudinal birth cohorts.^60,61^ Second, assessment of internalizing symptoms among offspring relied on 3 different questionnaires. Although they all measured the same construct, broadly defined as internalizing symptoms, measurement differences may have introduced bias. Relatedly, these measures required dichotomization to homogenize their distribution for our longitudinal model, which may have led to a loss of information. Nevertheless, the questionnaires used were appropriate for the specific age categories analyzed (ie, adolescence, early adulthood, and adulthood). Third, we assessed postnatal depression once and were therefore unable to account for variation in depressive symptoms in the first postnatal year. Fourth, we used a measure of depressive symptoms and not a clinical diagnosis, which prevents us from generalizing our results to clinical populations. Nevertheless, the Pitt inventory is a validated measure of depressive symptoms in general population samples that have relatively high prevalence of subclinical symptoms.

## Conclusions

This study, based on longitudinal data from South Africa, found that exposure to postnatal depression was associated with higher odds of internalizing symptoms among offspring persisting into adulthood, with important variations in odds increase by level of socioeconomic adversity and child sex. These findings stress the association between maternal and child emotional well-being, specifically that the emotional well-being of mothers is associated with the quality of children’s emotional development. This is true even in contexts, like that in this study, in which risk factors associated with mental health problems, including socioeconomic adversity, are widespread. Our findings suggest the need for further examination of the mechanisms of the association between maternal depression and offspring outcomes, including the interaction of socioeconomic adversity and child’s sex, in LMIC contexts. Such research may inform context-specific public policy aiming to promote maternal psychological well-being and child and adolescent development.

## Notes

Supplement. **eTable 1.** Fit Indices of Confirmatory Factor Analysis Model for Socioeconomic Adversity Index **eTable 2.** Marginal Probabilities for 3-Way Interaction Among Postnatal Depression, Socioeconomic Adversity, and Sex in the Association With Offspring Internalizing Symptoms **eFigure 1.** 3-Way Interaction Among Postnatal Depression, Socioeconomic Adversity, and Sex in the Association With Offspring Internalizing Problems **eFigure 2.** 3-Way Interaction Estimated Using Each Indicator Composing Socioeconomic Adversity Index
