# Supplementary material for: Analysis of Maternal Postnatal Depression, Socioeconomic Factors, and Offspring Internalizing Symptoms in a Longitudinal Cohort in South Africa
Source: JAMA Netw Open. 2021 Aug 19;4(8):e2121667. doi: 10.1001/jamanetworkopen.2021.21667 (PMC8377574; doi:10.1001/jamanetworkopen.2021.21667)
Supplement: Supplement. — eTable 1. Fit Indices of Confirmatory Factor Analysis Model for Socioeconomic Adversity Index eTable 2. Marginal Probabilities for 3-Way Interaction Among Postnatal Depression, Socioeconomic Adversity, and Sex in the Association With Offspring Internalizing Symptoms eFigure 1. 3-Way Interaction Among Postnatal Depression, Socioeconomic Adversity, and Sex in the Association With Offspring Internalizing Problems eFigure 2. 3-Way Interaction Estimated Using Each Indicator Composing Socioeconomic Adversity Index [file jamanetwopen-e2121667-s001.pdf]

Supplemental Online Content

Orri M, Besharati S, Ahun MN, Richter LM. Analysis of maternal postnatal depression, socioeconomic factors, and offspring internalizing symptoms in a longitudinal cohort in South Africa. *JAMA Netw Open*. 2021;4(8):e2121667. doi:10.1001/jamanetworkopen.2021.21667

eTable 1. Fit Indices of Confirmatory Factor Analysis Model for Socioeconomic Adversity Index
eTable 2. Marginal Probabilities for 3-Way Interaction Among Postnatal Depression, Socioeconomic Adversity, and Sex in the Association With Offspring Internalizing Symptoms
eFigure 1. 3-Way Interaction Among Postnatal Depression, Socioeconomic Adversity, and Sex in the Association With Offspring Internalizing Problemse
eFigure 2. 3-Way Interaction Estimated Using Each Indicator Composing Socioeconomic Adversity Index

This supplemental material has been provided by the authors to give readers additional information about their work.

© 2021 Orri M et al. *JAMA Network Open.*

eTable 1. Fit Indices of Confirmatory Factor Analysis Model for Socioeconomic Adversity Index

The Socioeconomic adversity index was calculated using a confirmatory factor analysis model with Mplus version 8 (weighted least square mean and variance adjusted estimator to take into account the binary nature of the indicator variables).

The fit indices of the model supports unidimensionality:[1]
Root Mean Square Error Of Approximation (RMSQA): 0.57
Comparative Fit Index (CFI): 0.955
Standardized Root Mean Square Residual (SRMR): 0.049

Standardized factor loading (all statistically significant and > 0.3) are as follows:

	Standardized factor loading	Standard error	z	P-Value
Poverty	0.458	0.043	10.68	< 0.001
Low maternal education	0.921	0.079	11.72	< 0.001
Low maternal age	0.347	0.046	7.506	< 0.001
Household crowding	0.318	0.036	8.754	< 0.001

1. Hu L, Bentler PM. Cutoff criteria for fit indexes in covariance structure analysis: Conventional criteria versus new alternatives. *Struct Equ Model Multidiscip J.* 1999;6(1):1-55. doi:10.1080/10705519909540118

© 2021 Orri M et al. *JAMA Network Open.*

eTable 2. Marginal Probabilities for 3-Way Interaction Among Postnatal Depression, Socioeconomic Adversity, and Sex in the Association With Offspring Internalizing Problems

We used set values of the continuous variables (mean, -1 SD below the mean, and +1 SD above the mean of postnatal depression and socioeconomic adversity z-scores) to estimate marginal probabilities and express the interaction in the probability scale. We tested whether the slopes where parallel with this parameterization (which is indicative of interaction in the additive scale) using the Delta method.

**Variables involved in the interaction**				**Delta-method**					
Postnatal depression Z score	Socioeconomic Adversity Z score	Sex		Margin	SE	z	*P* value	Lower 95%CI	Upper 95% CI
-1	-1	Female		0.07	0.02	2.92	0.003	0.02	0.11
-1	-1	Male		0.09	0.02	3.71	0.000	0.04	0.13
-1	0	Female		0.04	0.01	3.48	0.001	0.02	0.07
-1	0	Male		0.13	0.02	6.18	0.000	0.09	0.17
-1	1	Female		0.03	0.01	2.17	0.030	0.00	0.06
-1	1	Male		0.19	0.04	5.30	0.000	0.12	0.26
0	-1	Female		0.06	0.01	4.17	0.000	0.03	0.09
0	-1	Male		0.14	0.02	6.37	0.000	0.10	0.18
0	0	Female		0.05	0.01	5.21	0.000	0.03	0.07
0	0	Male		0.16	0.02	9.77	0.000	0.13	0.19
0	1	Female		0.04	0.01	3.35	0.001	0.02	0.06
0	1	Male		0.18	0.02	7.40	0.000	0.13	0.22
1	-1	Female		0.06	0.02	2.77	0.006	0.02	0.10
1	-1	Male		0.22	0.04	5.98	0.000	0.15	0.29
1	0	Female		0.06	0.01	4.30	0.000	0.03	0.09
1	0	Male		0.19	0.02	7.67	0.000	0.14	0.24
1	1	Female		0.06	0.02	3.25	0.001	0.02	0.09
1	1	Male		0.16	0.03	4.83	0.000	0.10	0.23

© 2021 Orri M et al. *JAMA Network Open.*

eFigure 1. 3-Way Interaction Among Postnatal Depression, Socioeconomic Adversity, and Sex in the Association With Offspring Internalizing Problems

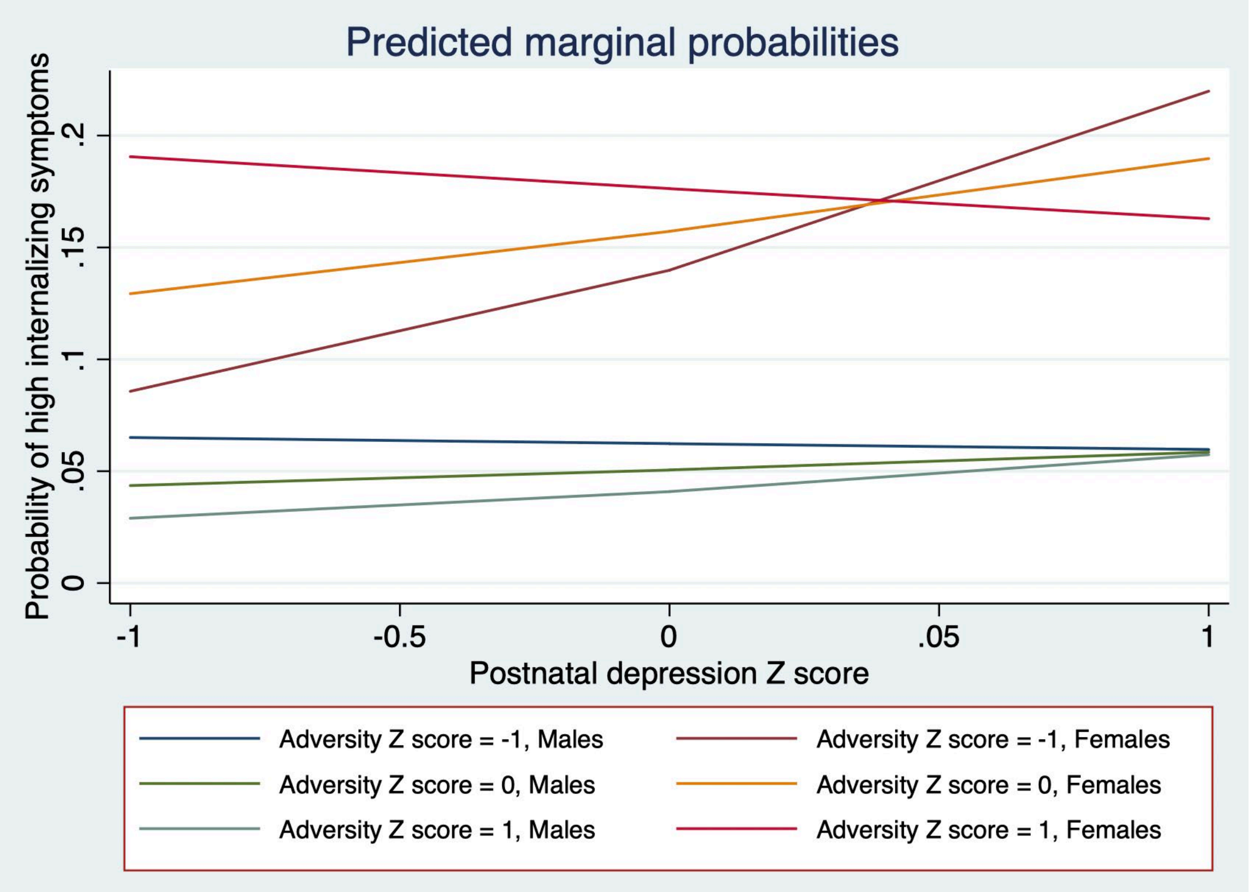

© 2021 Orri M et al. *JAMA Network Open.*

eFigure 2. 3-Way Interaction Estimated Using Each Indicator Composing Socioeconomic Adversity Index

The figure show that the interaction and direction of association for each indicator is consistent with the overall score.

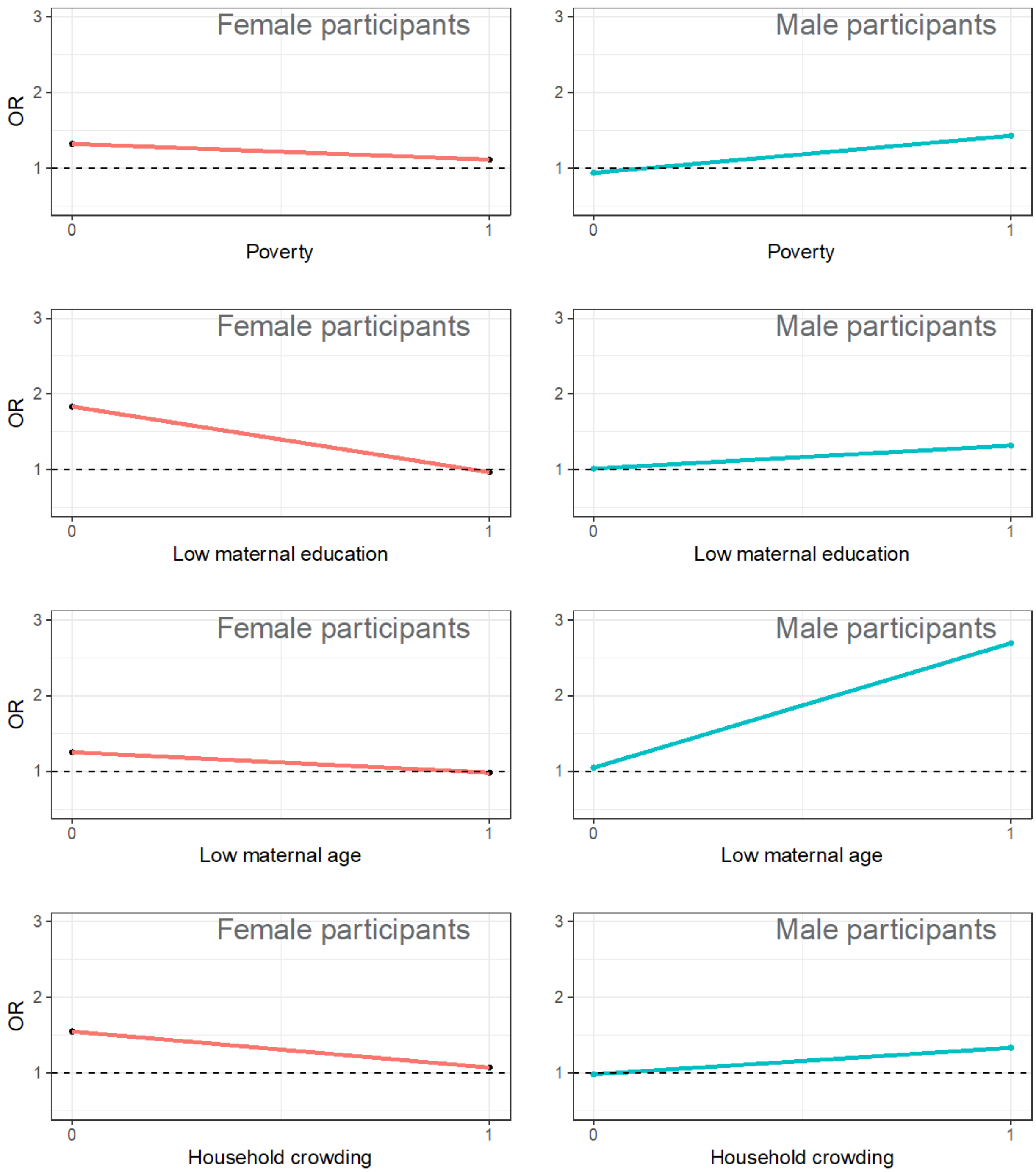

© 2021 Orri M et al. *JAMA Network Open.*
